# Absence of the lectin-like domain of thrombomodulin reduces HSV-1 lethality of mice with increased microglia responses

**DOI:** 10.1186/s12974-022-02426-w

**Published:** 2022-03-11

**Authors:** Meng-Shan Tsai, Li-Chiu Wang, Hua-Lin Wu, Shun-Fen Tzeng, Edward M. Conway, Sheng-Min Hsu, Shun-Hua Chen

**Affiliations:** 1 Institute of Basic Medical Sciences, College of Medicine, National Cheng Kung University, Tainan, Taiwan; 2 School of Medicine, I-Shou University, Kaohsiung, Taiwan; 3 Department of Biochemistry and Molecular Biology, College of Medicine, National Cheng Kung University, Tainan, Taiwan; 4 Department of Life Sciences, College of Biological Science and Biotechnology, National Cheng Kung University, Tainan, Taiwan; 5 Centre for Blood Research, Faculty of Medicine, Life Sciences Institute, University of British Columbia, Vancouver, BC Canada; 6 Department of Ophthalmology, College of Medicine, National Cheng Kung University, Tainan, Taiwan; 7 Department of Microbiology and Immunology, College of Medicine, National Cheng Kung University, Tainan, Taiwan

**Keywords:** HSV-1, Encephalitis, Thrombomodulin, Lectin-like domain, Microglia

## Abstract

**Background:**

Herpes simplex virus 1 (HSV-1) can induce fatal encephalitis. Cellular factors regulate the host immunity to affect the severity of HSV-1 encephalitis. Recent reports focus on the significance of thrombomodulin (TM), especially the domain 1, lectin-like domain (TM-LeD), which modulates the immune responses to bacterial infections and toxins and various diseases in murine models. Few studies have investigated the importance of TM-LeD in viral infections, which are also regulated by the host immunity.

**Methods:**

In vivo studies comparing wild-type and *TM-LeD* knockout mice were performed to determine the role of TM-LeD on HSV-1 lethality. In vitro studies using brain microglia cultured from mice or a human microglia cell line to investigate whether and how TM-LeD affects microglia to reduce HSV-1 replication in brain neurons cultured from mice or in a human neuronal cell line.

**Results:**

Absence of TM-LeD decreased the mortality, tissue viral loads, and brain neuron apoptosis of HSV-1-infected mice with increases in the number, proliferation, and phagocytic activity of brain microglia. Moreover, TM-LeD deficiency enhanced the phagocytic activity of brain microglia cultured from mice or of a human microglia cell line. Co-culture of mouse primary brain microglia and neurons or human microglia and neuronal cell lines revealed that TM-LeD deficiency augmented the capacity of microglia to reduce HSV-1 replication in neurons.

**Conclusions:**

Overall, TM-LeD suppresses microglia responses to enhance HSV-1 infection.

**Supplementary Information:**

The online version contains supplementary material available at 10.1186/s12974-022-02426-w.

## Introduction

Thrombomodulin (TM), a transmembrane glycoprotein in the C-type lectin-like domain superfamily, is widely expressed on the surface of various cells, including endothelial cells, leukocytes (monocytes, macrophages, and neutrophils), smooth muscle cells, and epithelial cells [1–4]. TM on endothelial cells binds thrombin to activate protein C, which inhibits both coagulation and inflammatory responses [2, 4–6]. TM is composed of five domains (domains 1–5) [4], and the domain 2 is responsible for thrombin binding and protein C activation [2, 4, 5, 7]. TM deficiency causes embryonic lethality in mice [8]. Recent reports focus on the domain 1, lectin-like domain (LeD), because *TM-LeD* knockout (TM^LeD/LeD^) mice with intact coagulation and protein C activation are viable [9], showing that TM-LeD deficiency fails to affect the known functions of TM in coagulation, protein C activation, and embryo development. TM^LeD/LeD^ mice are resistant to both Gram-positive and Gram-negative (Gram^−^) bacteria (*Streptococcus pneumonia* and *Burkholderia pseudomallei*) with reduced neutrophil and/or cytokine responses, when compared to wild-type (WT) mice [10, 11]. However, TM^LeD/LeD^ mice are more susceptible to treatments with Gram^−^ bacteria toxins, lipopolysaccharides (LPS) alone or LPS plus Shiga toxin, than WT mice [9, 12]. In experimental models with induced myocardial ischemia or arthritis, TM^LeD/LeD^ mice are prone to myocardial damage and inflammatory arthritis, when compared to WT mice [13, 14]. Elevated neutrophil, macrophage, monocyte, and/or cytokine responses are detected in TM^LeD/LeD^ mice treated with LPS or LPS plus Shiga toxin or stimulated to induce myocardial ischemia or arthritis [9, 12–14]. These studies show TM-LeD with immunomodulatory properties.

Herpes simplex virus (HSV) can induce encephalitis with an incidence of 1 in 200,000 individuals per year [15]. Acyclovir-related nucleoside analogs are used for patient treatment. The morality rates of encephalitis patients untreated or treated with acyclovir are over 70% and 30%, respectively, and only 2.5% of all encephalitis patients return to normal neurological function regardless treated with acyclovir or not [15, 16]. Herpes simplex virus 1 (HSV-1) and herpes simplex virus 2 (HSV-2) are two different species belonging to the *Simplexvirus* genus of *Herpesviridae* family [17]. HSV-1 infects the majority (> 80%) of human population worldwide [15, 18], accounts for more than 95% of encephalitis cases in patients beyond the neonatal period [19], and is often used to study encephalitis. Elevated levels of innate immune responses, microglia, monocytes, neutrophils, and interferons (IFNs) are detected in brains of encephalitis patients [20–22]. Although, TM-LeD regulates immune responses, which control viral infections, the significance of TM-LeD on viral infections remains elusive. By comparing WT and TM^LeD/LeD^ mice, we found that absence of TM-LeD reduces HSV-1 lethality with increases of brain microglia responses.

## Materials and methods

### Mice, cells, and viruses

The African green monkey kidney cell line (Vero), human neuroblastoma cell line (SK-N-SH), and human brain microglia cell line (HMC3) were maintained according to the instructions of the American Type Culture Collection. Both WT HSV-1 strains, KOS and 294.1, were isolated from patients with oral mucositis [23, 24]. The replication kinetics of KOS and 294.1 in vitro are comparable (data not shown). As the recombinant (VP16-GFP) HSV-1 strain (DG-1) [25] (kindly provided by Dr. David M. Knipe in Harvard Medical School) used for the in vitro assay of HSV-1 entry into mouse primary brain microglia was constructed from KOS [26]. For the consistence of in vitro studies, KOS was used. In C57BL/6J mice, which were used to generate TM^LeD/LeD^ mice, 294.1 can induce death and was used for study. Viruses were propagated and titrated on Vero cell monolayers by the plaque assay. Briefly, virus in solution was inoculated onto the Vero cell monolayer seeded the day before. After incubation for 45 min (min) at room temperature, the infected cell monolayer was overlaid with medium containing 1% methylcellulose, cultured for 3 days, and stained with crystal violet to detect plaques. C57BL/6J mice were purchased from the National Laboratory Animal Center of Taiwan. C57BL/6J-derived TM^LeD/LeD^ mice constructed by targeted disruption of the gene [9] were bred under specific-pathogen-free conditions in the laboratory animal center of our college.

### Infection of mice, depletion of microglia in mice, and collection of mouse tissues

Six-to-eight-week-old female mice were weighted, anesthetized with pentobarbital sodium (65 mg/kg), and inoculated with HSV-1 (1 × 10^7^ PFU/eye) topically on one eye after scarification of cornea with a 26G needle for 10–12 times. Infected mice were monitored for survival and body weights. Mice were euthanized by injecting over dose of pentobarbital sodium (> 90 mg/kg) when the mouse body weights were below 20% of those on the day of infection according to the mouse experiment protocols approved by the Institutional Animal Care and Use Committee (IACUC) of National Cheng Kung University with the approval numbers of 108063 and 109293. Euthanized mice were counted as dead. To deplete microglia, mice were fed with chow containing PLX5622 (kindly provided by Plexxikon Inc.) at the dose of 1200 mg/kg chow [27, 28] from 4 weeks to the end of experiment. PLX5622-treated mice were infected at 6 weeks. The infected eyes, ipsilateral trigeminal ganglia of infected eyes, and whole brains of infected mice were harvested, homogenized, and assayed for virus titers by plaque assay.

### Immunofluorescence staining

Mice were anesthetized by sodium pentobarbital (65 mg/kg) and perfused intracardially with phosphate-buffered saline (PBS) and 4% paraformaldehyde. Brains were harvested, fixed in 4% paraformaldehyde at 4 °C overnight and then in 30% sucrose in PBS for 2–3 days, embedded in OCT (Thermo Fisher Scientific), and frozen. Coronal brain sections (30 μm) were prepared by the sliding freezing microtome (Leica SM 2010R). Sections were immersed in 10 mM sodium citrate buffer (pH 6), heated in boiling water for 15 min, and incubated with blocking and permeabilization buffer (PBS, 5% fetal bovine serum, and 0.1% Triton X-100) for 1 h at room temperature. Cells in culture were fixed in 4% paraformaldehyde for 20 min at room temperature, washed three times with PBS, and incubated with blocking and permeabilization buffer for 1 h at room temperature. The resulting brain sections or cells were incubated with primary antibodies against mouse NeuN (clone A60; Merck Millipore or clone D4G4O; Cell Signaling), HSV-1 (Dako), cleaved caspase 3 (Cell Signaling), Iba1 (Synaptic Systems or GeneTex), or MAP2 (Abcam) at 4 °C overnight. Subsequently, the samples were incubated with Alexa Fluor-conjugated secondary antibodies (Thermo Fisher Scientific or Jackson ImmunoResearch Laboratories, Inc.) in blocking and permeabilization buffer for 2 h at room temperature and mounted in mounting medium with DAPI (Abcam), which stains the nucleus. Images were photographed by the confocal laser scanning microscope, FV3000 (IX83; Olympus) with 10× (0.4 NA), 20× (0.75 NA), 40× (1.3 NA), 60× (1.35 NA), and 100× (1.4 NA) objectives (Olympus) and the FV31-HSD scanner. Acquired images were quantified by the software of ImageJ (National Institutes of Health, USA) and analyzed by the Pearson correlation coefficient.

### Flow cytometry

Mouse brains were harvested and dissociated, and leukocytes were isolated and blocked with the antibody CD16/CD32 (Clone 93; BioLegend) to prevent nonspecific binding as previously described [29] before staining with antibodies against mouse leukocyte antigens, CD45 (Clone 30F11; BD Biosciences), CD11b (Clone M1/70; BD Biosciences), Tmem119 (Clone 106-6; Abcam), MHC-II (Clone I-A/I-E; BD Biosciences), CD86 (Clone GL-1; BioLegend), Ly6G (Clone 1A8; BD Biosciences), CD3 (Clone 145-2C11; BD Biosciences), or CD68 (Clone FA-11; BioLegend). For detection of cell viability, cells were stained with the fixable viability dye (Invitrogen) for 30 min on ice in the dark. For detection of Ki67^+^ cells, cells were fixed and permeabilized by Cytofix/Cytoper (BD Biosciences) before incubation with the anti-Ki67 antibody (Clone 16A8; BioLegend). The stained cells were analyzed by the flow cytometer, CantoII (BD Biosciences) and analyzed with the FlowJo software (Treestar Inc.).

### Culture of mouse primary cells

Neurons were isolated from the cortex and hippocampus of mouse embryos at 18.5 day post-coitum as previously described [30, 31]. Briefly, mouse cortical tissues were digested with papain (Sigma-Aldrich) for 15–20 min at 37 °C and dissociated into single cells in Neurobasal medium (Invitrogen, Thermo Fisher Scientific) containing 2 mM l-glutamine and 2% B-27 (Invitrogen, Thermo Fisher Scientific) by pipetting. After centrifugation, cells were seeded onto 24-well plates coated with poly-d-lysine (Invitrogen, Thermo Fisher Scientific) with Neurobasal medium at a density of 1 × 10^5^ cells per well. Primary neurons cultured for 5 days were used for experiments. Microglia were isolated from the mouse cortex at postnatal day 1 or 2 as previously described [32]. Briefly, mouse cortices were dissociated and suspended in DMEM/F12 medium containing 10% fetal bovine serum. Cells were then cultured on flasks coated with poly-d-lysine. After 12–14 days, microglia were detached from mixed glial culture by the shake-off method and seeded onto 24-well plates coated with poly-d-lysine. The purity of neurons and microglia was > 95% based on the observation of cell morphology.

### Microglia phagocytosis assay

Mouse primary microglia and HMC3 cells were subjected to the phagocytosis assay with the commercially available kit (BioVision) according to the instructions of manufacturer. Briefly, cells were incubated with green fluorescence-labeled zymosan for 1 h at 37 °C, washed, and resuspended in the quenching solution for 2 min at room temperature to destroy (quench) fluorophores outside cells. The resulting cells were analyzed by the flow cytometer, CantoII (BD Biosciences) and FlowJo software (Treestar Inc.).

### Entry of HSV-1 into microglia

Mouse primary microglia were infected with recombinant (VP16-GFP) HSV-1 strain (DG-1) at a multiplicity of infection (MOI) of 10 at 37 °C for 30 min, incubated with citrate buffer (pH 3) at room temperature for 1 min to remove virus outside cells, and fixed with 4% paraformaldehyde for 20 min. The fluorescence intensity of cells was detected by the multi-mode microplate reader, SpectraMax iD3 (Molecular Devices).

### Establishment of the HMC3 cell line with TM-LeD deficiency by the CRISPR/Cas9 system

HMC3 cells with TM-LeD deficiency were generated by the CRISPR/Cas9 system using gRNAs, 5′-GCGCCTGGGTAACATGCTT-3′ and 5′-TGTGCCCAGCGAGCCGATC-3′ (National C6 RNAi Core Facility at the Academia Sinica in Taiwan), to target and remove the 465-base-pair coding sequence. Briefly, cells were transfected with the CRISPR plasmid using Lipofectamine® 3000 (Thermo). After 24 h, the transfection medium was replaced with culture medium containing 1 μg/ml of puromycin and cultured for 7 days to select edited cells. The resulting cells were harvested, processed for extraction of genomic DNA using the QIAamp® DNA mini kit (QIAGEN), and subjected to PCR analysis with forward (5′-GGAGGACCAAGAG ATGAAAGAGGGCTG-3′) and reverse (5′-GAACGCAGAAGTGCTCGCAGAGGTC-3′) primers, which generated 1379- and 929-base-pair products for WT and *TM-LeD*-deleted cells, respectively. In the sample with *TM-LeD*-deleted cells (with both 1379- and 929-base-pair PCR products), cells were diluted and plated on 96-well to obtain the single cell clone, which was cultured and subjected to PCR analysis to confirm the genotype. Cell proliferation was monitored by microscope observation or the cell counting kit-8 (Enzo Life Sciences) according to the instructions of manufacture.

### Western blotting

Cells were washed with PBS, frozen at − 80 °C, and incubated with cell lysis buffer (Cell Signaling) for 30 min. Total proteins were extracted from cells, measured for concentrations using the protein assay dye reagent concentrate (Bio-Rad) according to the instructions of manufacture, separated by SDS–PAGE, and transferred onto nitrocellulose membranes (Merck Millipore). Blots were blocked with 5% skim milk to prevent nonspecific binding and stained with antibodies against CD68 (Clone FA-11; Abcam) or β-actin (Clone AC-15; Sigma-Aldrich) at 4 °C overnight and incubated with corresponding horseradish peroxidase-conjugated secondary antibodies for chemiluminescence detection. Images were detected and photographed by the UVP BioSpectrum AC imaging system and analyzed by the ImageJ software.

### Statistical analyses

Data represent means ± SEM (error bar) values. Data of Kaplan–Meier survival curves were analyzed by the log-rank test. Data of virus titers were analyzed by the Mann–Whitney *U* test. Data of in vivo flow cytometry analyses (in Figs. 2 and 3) were analyzed by the Mann–Whitney *U* test. Results of Fig. 4, markers of microglia phagocytosis (CD45^int^CD11b^+^CD68^+^), were analyzed the two-way ANOVA with consideration of the timing effect. The rest of data were analyzed by the Student *t* test. The statistics were calculated using GraphPad Prism 6.0. A *P* value of < 0.05 is considered statistically significant.

## Results

### Absence of TM-LeD decreases HSV-1 lethality in mice with reduced tissue viral loads

**Fig. 1:**
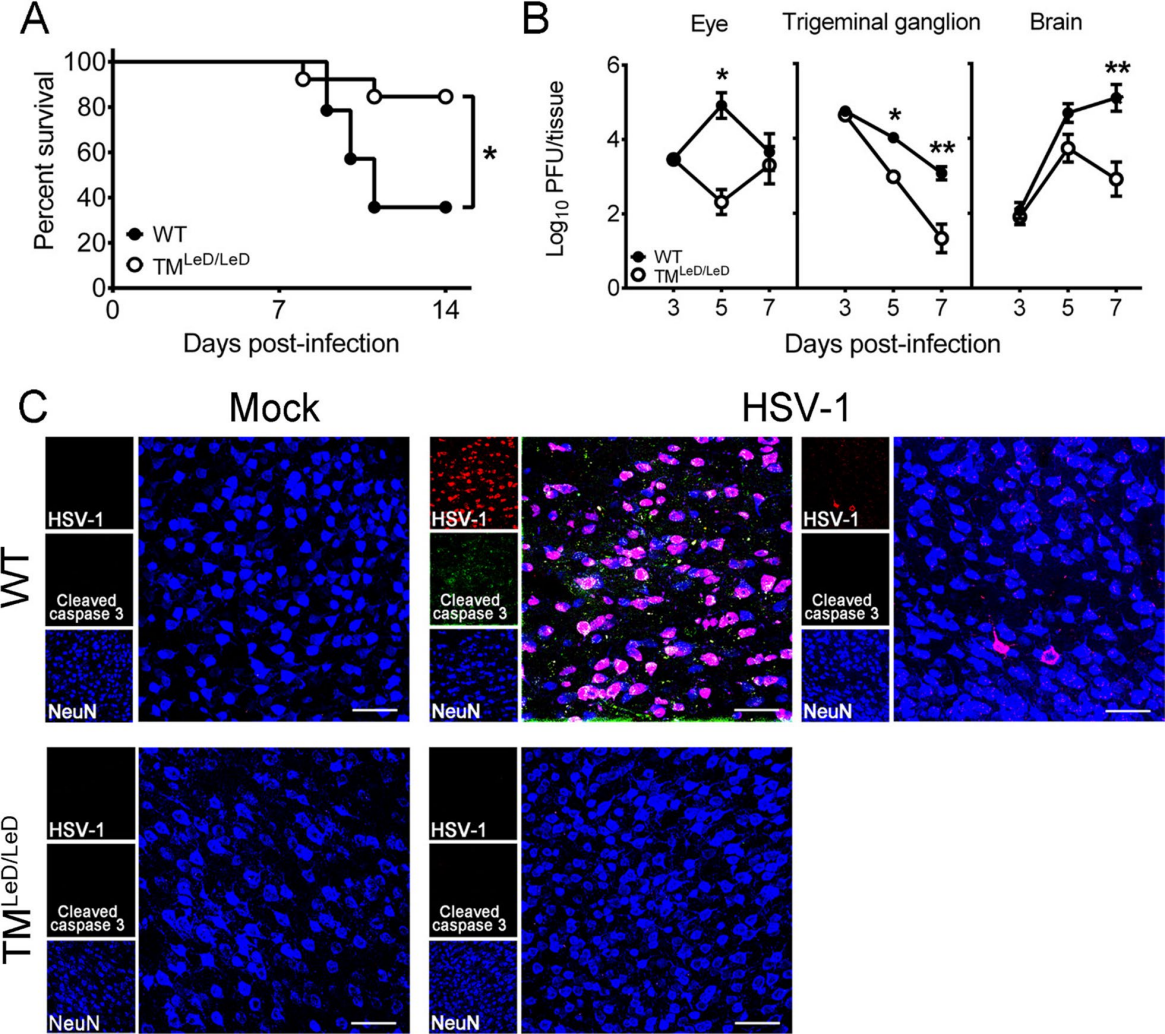
Absence of TM-LeD reduces mortality, tissue viral loads, and brain neuron apoptosis of HSV-1-infected mice. **A** The survival rates of infected WT mice (*n* = 14) and TM^LeD/LeD^ mice (*n* = 13) are shown. **B** Virus titers in the eyes, trigeminal ganglia, and brains of WT and TM^LeD/LeD^ mice on the indicated dpi are shown. The data represent means ± SEM (error bars) of 5–12 sample per data point. *P < 0.05 and **P < 0.01. **C** Sections of brains harvested from mock-infected or infected WT or TM^LeD/LeD^ mice at 5 dpi were subjected to immunofluorescence staining with antibodies against HSV-1 and mouse cleaved caspase 3 and NeuN. The temporal cortex region is shown. Scale bar, 50 μm. Data are representative of at least three samples per group from two independent experiments

To study the effect of TM-LeD on HSV-1 infection in vivo, we monitored the survival rates of WT and TM^LeD/LeD^ mice infected with the virus on the scarified cornea, a site in humans that can be infected by the virus [15]. The survival rates of infected female WT and TM^LeD/LeD^ mice were 85% (11 of 13) and 36% (5 of 14), respectively, by 14 day post-infection (dpi) (Fig. 1A). Because the survival rates of infected male WT mice (50%, 2 of 4) and TM^LeD/LeD^ mice (50%, 3 of 6) were comparable, female mice were used for studies. In our infection model, HSV-1 was detected in the brain, trigeminal ganglia, and eye, but not in other tissues, organs, or blood of mice. We monitored viral loads and found that the virus titers in these tissues of WT mice were higher than those of TM^LeD/LeD^ mice at 5 and/or 7 dpi (Fig. 1B).

We also monitored mouse brains for HSV-1 antigens and for damage using cleaved caspase 3, a marker of apoptosis, by immunoflourescence staining. The brain virus titers of infected TM^LeD/LeD^ mice reached a peak at 5 dpi (Fig. 1B), so we performed staining of HSV-1 antigens with focus at this timepoint. Both viral antigens and cleaved caspase 3 were below detection in brains of mock-infected WT and TM^LeD/LeD^ mice (Fig. 1C). In the brain of infected WT mice, HSV-1 antigens were detected mostly in the cells positive for NeuN, a marker of neuron nuclei, in the temporal cortex, in which the abundance of viral antigens varied between different regions (Fig. 1C). In the temporal cortex, cleaved caspase 3 was detected and colocalized with HSV-1 antigens in NeuN^+^ cells in the region with abundant viral antigens, but was below detection in the region with few HSV-1 antigens (Fig. 1C), in a manner correlated with the viral antigen level. Few HSV-1 antigens and cleaved caspase 3 were detected in other brain regions, such as frontal cortex, olfactory bulbs, hippocampus, brain stem, and cerebellum (data not shown). Our results showing cleaved caspase 3 and HSV-1 antigens in the temporal cortex are consistent with those found in HSV-1 encephalitis patients with destruction and viral antigens detected in the brain temporal cortex [33]. In infected WT mice, HSV-1 antigens and cleaved caspase 3 were below detection in all mouse brain regions at 3 dpi and were not significantly increased in the temporal cortex at 7 dpi when compared to 5 dpi (data not shown). In infected TM^LeD/LeD^ mice, both cleaved caspase 3 and HSV-1 antigens were below detection in all mouse brain regions from 3 to 7 dpi with the result of the temporal cortex at 5 dpi shown in Fig. 1C. The cleaved caspase 3 result suggests that HSV-1 infection induces brain neuron damage in (WT) mice and that absence of TM-LeD reduces the brain neuron damage of HSV-1-infected mice.

As elevated tissue viral loads were detected in WT mice, we, therefore, investigated the effect of TM-LeD on HSV-1 replication using mouse brain neurons, embryonic fibroblasts (MEFs), and brain microglia cultured from WT or TM^LeD/LeD^ mice (Additional file 3: Fig. S1). We found that brain neurons and MEFs supported HSV-1 replication and produced high virus titers when the cells were infected with a low MOI (0.001) of virus. However, virus titers were below detection in brain microglia infected with HSV-1 at MOIs of ≤ 0.1 (data not shown). Microglia still failed to support viral replication and reduced virus infectivity when the cells were infected with virus at the MOI of 1 (Additional file 3: Fig. S1). Comparable virus titers were detected in WT and TM^LeD/LeD^ cells of all three cell types, indicating that TM-LeD deficiency might suppress HSV-1 replication in cells indirectly.

### Absence of TM-LeD increases the number and proliferation of microglia in brains of HSV-1-infected mice

We previously reported that exposure of TM^LeD/LeD^ mice to LPS resulted in an increase in lung neutrophil infiltration [9]. We, therefore, assessed the effect of TM-LeD on brain leukocyte responses in HSV-1-infected mice with focus on neutrophils, macrophages, and T cells, which are shown to infiltrate into brains during infection [34, 35]. Flow cytometric results showed that in mock-infected WT and TM^LeD/LeD^ mice, the numbers of (CD45^hi^) infiltrating myeloid cells, (CD45^hi^CD11b^+^Ly6G^−^) macrophages and (CD45^hi^CD11b^+^Ly6G^+^) neutrophils, were low (Additional file 4: Fig. S2A, B). In infected WT and TM^LeD/LeD^ mice, the numbers of infiltrating macrophages and neutrophils were comparable 3–7 dpi (Additional file 4: Fig. S2A, B). In mock-infected and infected WT and TM^LeD/LeD^ mice, the numbers of (CD45^hi^CD3^+^) T cells, which includes CD4 T and CD8 T cells, were low and comparable at 5 dpi (Additional file 4: Fig. S2C). These results were consistent with previous reports including ours showing that (CD3, CD4, and CD8) T cells infiltrate into brains of HSV-1-infected mice at 7 dpi, but not at 5 dpi [34, 36, 37]. We, therefore, monitored CD4 and CD8 T cells at 7 dpi. In mock-infected WT and TM^LeD/LeD^ mice, the numbers of (CD45^hi^CD4^+^) CD4 T cells and (CD45^hi^CD8^+^) CD8 T cells were low and comparable (Additional file 4: Fig. S2D, G). In infected mice, the number of CD4 T cells in WT mice was higher than that of TM^LeD/LeD^ mice (Additional file 4: Fig. S2D). Further analyses of CD4 T cells showed that the numbers of (CD45^hi^CD4^+^Foxp3^+^) regulatory T cells and T_H_2 (CD45^hi^CD4^+^GATA3^+^) cells in infected WT mice were significantly and slightly higher than those of infected TM^LeD/LeD^ mice (Additional file 4: Fig. S2E, F). The numbers of CD8 T cells and activated CD8 (CD45^hi^CD8^+^CD160^+^) T cells in infected WT and TM^LeD/LeD^ mice were comparable (Additional file 4: Fig. S2G, H). Collectively, absence of TM-LeD decreases the infiltration of CD4 T cells, especially regulatory T cells, but not myeloid or CD8 T cells, into brains of infected mice.

Reports of others and ours show that microglia play a protective role in HSV-1 infection [34, 35, 38–41]. Our recent report found that microglia depletion increases HSV-1 lethality in mice with elevated brain levels of neutrophils, CD4 T cells, CD8 T cells, IFN-β, and IFN-γ, which are futile to reduce brain viral loads, showing the significance of microglia in HSV-1 infection [34]. Prior to evaluating the effect of TM-LeD on microglia, we examined TM expression in microglia of WT mice using immunofluorescence staining, as the antibody against TM-LeD is unavailable commercially. TM was detected in about 35% cells positive for Iba1, a marker of microglia, but not in NeuN^+^ cells (data not shown), in brains of mock-infected WT mice.

**Fig. 2:**
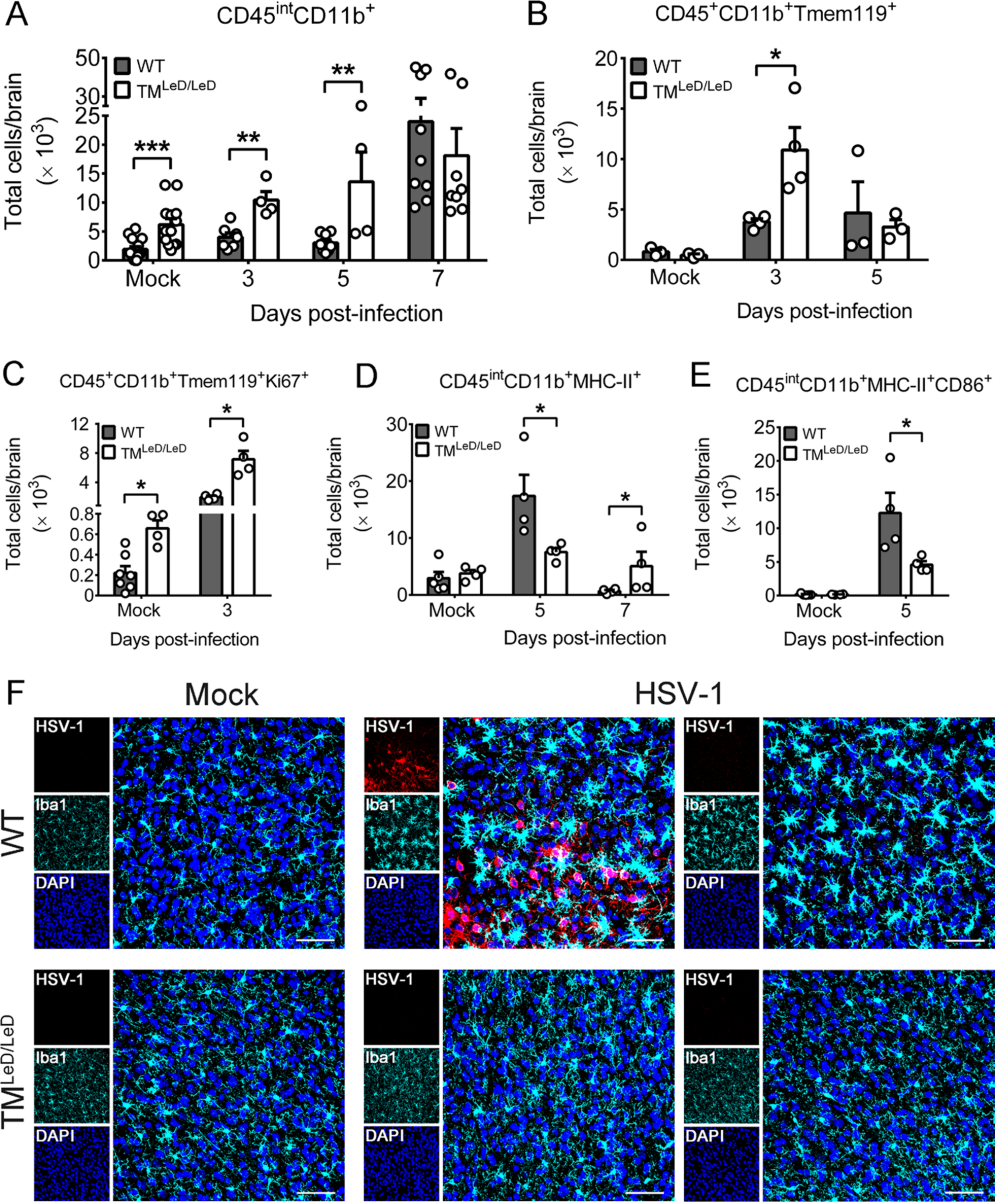
Effects of TM-LeD on the number, proliferation, and activation of brain microglia in HSV-1-infected mice. Leukocytes purified from brains of WT or TM^LeD/LeD^ mice mock-infected (Mock) or infected with HSV-1 at the indicated dpi were assayed for markers of **A** microglia (CD45^int^CD11b^+^), **B** microglia (CD45^+^CD11b^+^Tmem119^+^), **C** microglia undergoing proliferation (CD45^+^CD11b^+^Tmem119^+^Ki67^+^), and activated microglia, CD45^int^MHC-II^+^CD11b^+^ (**D**) or CD45^int^MHC-II^+^CD11b^+^CD86^+^ (**E**) by flow cytometry. The data represent means + SEM (error bars) of 4–14 samples per group. *P < 0.05; **P < 0.01; and ***P < 0.001. **F** Sections of brains harvested from mock-infected or infected WT or TM^LeD/LeD^ mice at 5dpi were subjected to immunofluorescence staining with DPAI for the nucleus and the antibody against HSV-1 or mouse Iba1. The temporal cortex region is shown. Image data are representative of at least three samples per group from two independent experiments. Scale bar, 50 μm

We further quantified brain microglia using flow cytometry. In mock-infected mice, the numbers of (CD45^int^CD11b^+^) microglia were low, especially in WT mice, when compared to TM^LeD/LeD^ mice, with 3.2-fold difference between two mouse groups (Fig. 2A). After infection, the numbers of microglia in WT mice were lower than those of TM^LeD/LeD^ mice at 3 and 5 dpi by 2.5- and 4.5-fold, respectively (Fig. 2A). Tmem119 is another marker detected predominantly in microglia [42]. In mock-infected mice, the numbers of (CD45^+^CD11b^+^Tmem119^+^) microglia were low and comparable in WT and TM^LeD/LeD^ mice (Fig. 2B). After infection, the number of (CD45^+^CD11b^+^Tmem119^+^) microglia in WT mice was lower than that of TM^LeD/LeD^ mice at 3 dpi, but not at 5 dpi (Fig. 2B). To address whether the abundant (CD45^+^CD11b^+^Tmem119^+^) microglia detected in infected TM^LeD/LeD^ mice at 3 dpi (Fig. 2B) are due to proliferation, we assessed Ki67, a cell proliferation marker, at this timepoint. The numbers of (CD45^+^CD11b^+^Tmem119^+^Ki67^+^) microglia in mock-infected and infected TM^LeD/LeD^ mice were higher than those of WT mice by 2.9- and 3.8-fold, respectively (Fig. 2C). Absence of TM-LeD promotes microglia proliferation in mock-infected and infected mice (Fig. 2C). Collectively, absence of TM-LeD increases the number and proliferation of brain microglia in mock-infected and infected mice.

Microglia are antigen-presenting cells. As an increase of brain CD4 T cells, which is stimulated by antigen-presenting cells, was detected in infected WT mice at 7 dpi (Additional file 4: Fig. S2D), we, therefore, assessed the antigen-presenting activities of brain microglia with the activation marker (MHC-II) and co-stimulating molecule marker (CD86) at 7 dpi and the early timepoint, 5 dpi. The number of CD45^int^MHC-II^+^CD11b^+^ microglia in infected WT mice was higher than that of infected TM^LeD/LeD^ mice at 5 dpi, but not at 7 dpi (Fig. 2D). At 5 dpi, the number of CD45^int^MHC-II^+^CD11b^+^CD86^+^ microglia with capacity to stimulate CD4 T cells in infected WT mice was also higher than that of infected TM^LeD/LeD^ mice (Fig. 2E). In mock-infected WT and TM^LeD/LeD^ mice, the numbers of CD45^int^MHC-II^+^CD11b^+^ and CD45^int^MHC-II^+^CD11b^+^CD86^+^ microglia were low and comparable (Fig. 2D, E).

Microglia undergo morphologic changes with a ramified shape during resting and surveying and with an amoeboid or round shape during activation [43, 44]. Activated microglia display an enlarged cell body and short processes with reduced branching. To assess the effect of TM-LeD on microglia activation by assessing the cell morphology using immunofluorescence staining, we monitored the cells positive for Iba1, activated microglia, in mouse brains at 5 dpi, when microglia of infected WT mice showed a higher level of activation marker (MHC-II) than those of infected TM^LeD/LeD^ mice (Fig. 2D). Microglia of mock-infected WT and TM^LeD/LeD^ mice displayed a ramified shape in all brain regions with the region of temporal cortex shown in Fig. 2F. Microglia of infected WT mice displayed the activated (amoeboid or round) shape with enlarged cell body and short processes and branching in all brain regions regardless the levels of HSV-1 antigens with the temporal cortex region shown in Fig. 2F. Microglia of infected TM^LeD/LeD^ mice displayed the ramified shape with reduced cell body in all brains regions with the temporal cortex region shown in Fig. 2F.

TM-LeD hijacks the high mobility group box 1 (HMGB1) to suppress the receptor for advanced glycation end-products (RAGE) and downstream signaling, NF-κB and/or MAPK, of non-microglia after LPS challenge [2, 13, 45, 46]. Moreover, activated microglia can produce cytokines to fight viral infections. We measured the pro-inflammatory cytokines, IL-1β and TNF-α, which are regulated by NF-κB and MAPK, can be produced by microglia, and are reported protect mice against HSV-1 [47], in brains of mice at 5 and 7 dpi, because microglia of infected WT mice showed a higher level of activation than those of infected TM^LeD/LeD^ mice at 5 dpi (Fig. 2D, F). The protein levels of IL-1β and TNF-α in the brains of infected WT and TM^LeD/LeD^ mice were comparable at 5 and 7 dpi (Additional file 5: Fig. S3A), suggesting that TM-LeD increases HSV-1 lethality in mice by the mechanism likely independent of the HMGB1–RAGE–NF-κB and/or MAPK pathways. In addition, the protein levels of antiviral cytokines, type I IFNs (IFN-α and IFN-β) and type II IFN (IFN-γ) which protect mice against HSV-1 [48–50], in the brains of infected WT and TM^LeD/LeD^ mice at 7 and/or 5 dpi were comparable (Additional file 5: Fig. S3A).

### Microglia depletion renders TM^LeD/LeD^ mice susceptible to HSV-1 infection with increased mortality and tissue viral loads

**Fig. 3:**
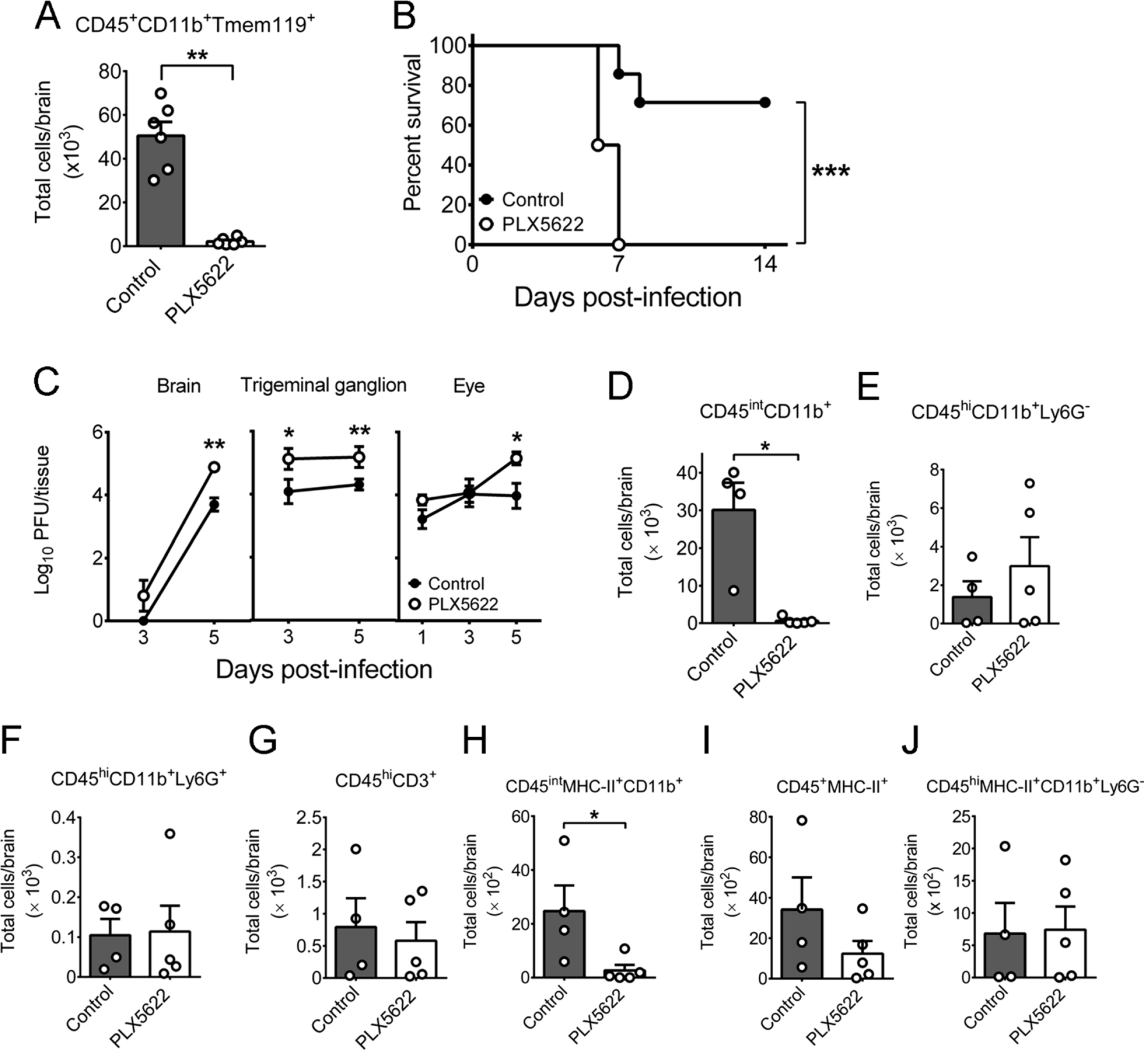
Microglia depletion increases HSV-1 lethality and tissue viral loads of TM^LeD/LeD^ mice. **A** The numbers of microglia in mice treated without (Control) or with PLX5622 right before infection are shown. **B** The survival rates of TM^LeD/LeD^ mice fed with control chow (Control; *n* = 7) or chow containing PLX5622 (PLX5622; *n* = 8) and infected with HSV-1 are shown. **C** Virus titers in the indicated mouse tissues are shown. Leukocytes purified from brains of infected mice at 5 dpi were assayed for markers of **D** microglia (CD45^int^CD11b^+^), **E** macrophages (CD45^hi^CD11b^+^Ly6G^−^), **F** neutrophils (CD45^hi^CD11b^+^Ly6G^+^), **G** T cells (CD45^hi^CD3^+^), **H** activated microglia (CD45^int^MHC-II^+^CD11b^+^), **I** activated myeloid cells (CD45^+^MHC-II^+^), or **J** activated macrophage (CD45^hi^MHC-II^+^CD11b^+^Ly6G^−^) by flow cytometry. The data represent means ± or + SEM (error bars) of 5–8 samples per data point in panel **C** and 4–6 samples per group in panels **A** and **D**–**J**. *P < 0.05; **P < 0.01; and ***P < 0.001

Increases of brain microglia and survival rate were found in infected TM^LeD/LeD^ mice (Figs. 1A, 2A). To determine whether microglia protect TM^LeD/LeD^ mice against HSV-1, we depleted microglia by feeding the mice with chow containing the compound, PLX5622, which blocks the signaling of colony-stimulating factor 1 receptor needed for microglia survival, growth, and proliferation [51, 52]. Treatment with PLX5622 2 weeks prior to infection reduced (CD45^+^CD11b^+^Tmem119^+^) microglia by 95% in the mouse brain when compared to the control chow as demonstrated by flow cytometry (Fig. 3A). After feeding with chow for 2 weeks, the mice were infected with HSV-1 and fed with chow with or without PLX5622. The mortality of PLX5622-treated mice was 100% (8 of 8), which was higher than that of control mice (29%, 2 of 7), at 14 dpi (Fig. 3B). The virus titers in the brains, trigeminal ganglia, and eyes of the PLX5622-treated mice were higher than those of control mice at 5 and/or 3 dpi (Fig. 3C). At 5dpi, but not 3 dpi, the brain virus titers of PLX5622-treated and control mice were significantly different (Fig. 3C). At 7 dpi, all PLX5622-treated mice infected with virus succumbed to death (Fig. 3B). We, therefore, monitored mouse brains at 5 dpi for leukocyte levels and activities. PLX5622 treatment reduced the number of (CD45^int^CD11b^+^) microglia by 95% and the level of antigen-presenting activity of microglia (CD45^int^MHC-II^+^CD11b^+^) in the brain (Fig. 3D, H). The numbers of (CD45^hi^CD11b^+^Ly6G^−^) macrophages, (CD45^hi^CD11b^+^Ly6G^+^) neutrophils, (CD45^hi^CD3^+^) T cells, (CD45^+^MHC-II^+^) activated myeloid cells, and (CD45^hi^MHC-II^+^CD11b^+^Ly6G^−^) activated macrophages in brains of infected mice treated with or without PLX5622 were statistically insignificant (Fig. 3E–G, I, J), showing that PLX5622 treatment failed to affect the numbers and activation of infiltrating leukocytes in the brains of infected mice.

### Absence of TM-LeD enhances the phagocytic activity of brain microglia during HSV-1 infection as demonstrated by mouse in vivo and in vitro studies

As microglia protect infected TM^LeD/LeD^ mice with decreases of tissue viral loads, we assessed how TM-LeD affects the antiviral action of microglia. Activated microglia can produce antiviral cytokines, IFN-β and IFN-γ [40, 53], which can reduce HSV-1 replication by activating (phosphorylating) the transcription factor, signal transducer and activator of transcription (STAT) 1, of interferon-stimulated genes (ISGs) [54, 55]. We, therefore, monitored the expression (mRNA levels) of antiviral mediators, *Ifnb*, *Ifng*, *Ifnl* and ISGs, *Cxcl10* and *MX dynamin-like GTPase 1* (*Mx1*), in microglia isolated from brains at 5 dpi, when microglia of infected WT mice showed a higher level of activation than those of infected TM^LeD/LeD^ mice at 5 dpi (Fig. 2D, F). Quantitative RT-PCR results showed that the mRNA levels of these antiviral mediators in WT and TM^LeD/LeD^ microglia were comparable (Additional file 5: Fig. S3B). We observed reduced antigen-presenting activities of microglia in the brains of infected TM^LeD/LeD^ mice at 5 dpi (Fig. 2D, E), prompting us to investigate whether TM-LeD regulates microglia for M1/M2 transition, which affects the antigen presentation activity [56]. This could be achieved by monitoring the gene expression of M1-associated factors (pro-inflammatory modulators), *Nos2*, *Il1b*, *Il6*, and *Tnf*, and M2-associated factors (anti-inflammatory modulators), *Arg1*, *Il10*, and *Tgfb*, by quantitative RT–PCR (Additional file 1 and Additional file 2: Table S1). The mRNA levels of M1- and M2-associated factors in microglia isolated from the brains of infected WT or TM^LeD/LeD^ mice at 5 dpi were statistically insignificant (Additional file 5: Fig. S3B). Proteins and mRNA levels of IFN-β, IFN-γ, IL-1β, and TNF-α in brains and brain microglia of infected WT or TM^LeD/LeD^ mice were comparable (Additional file 5: Fig. S3B).

**Fig. 4:**
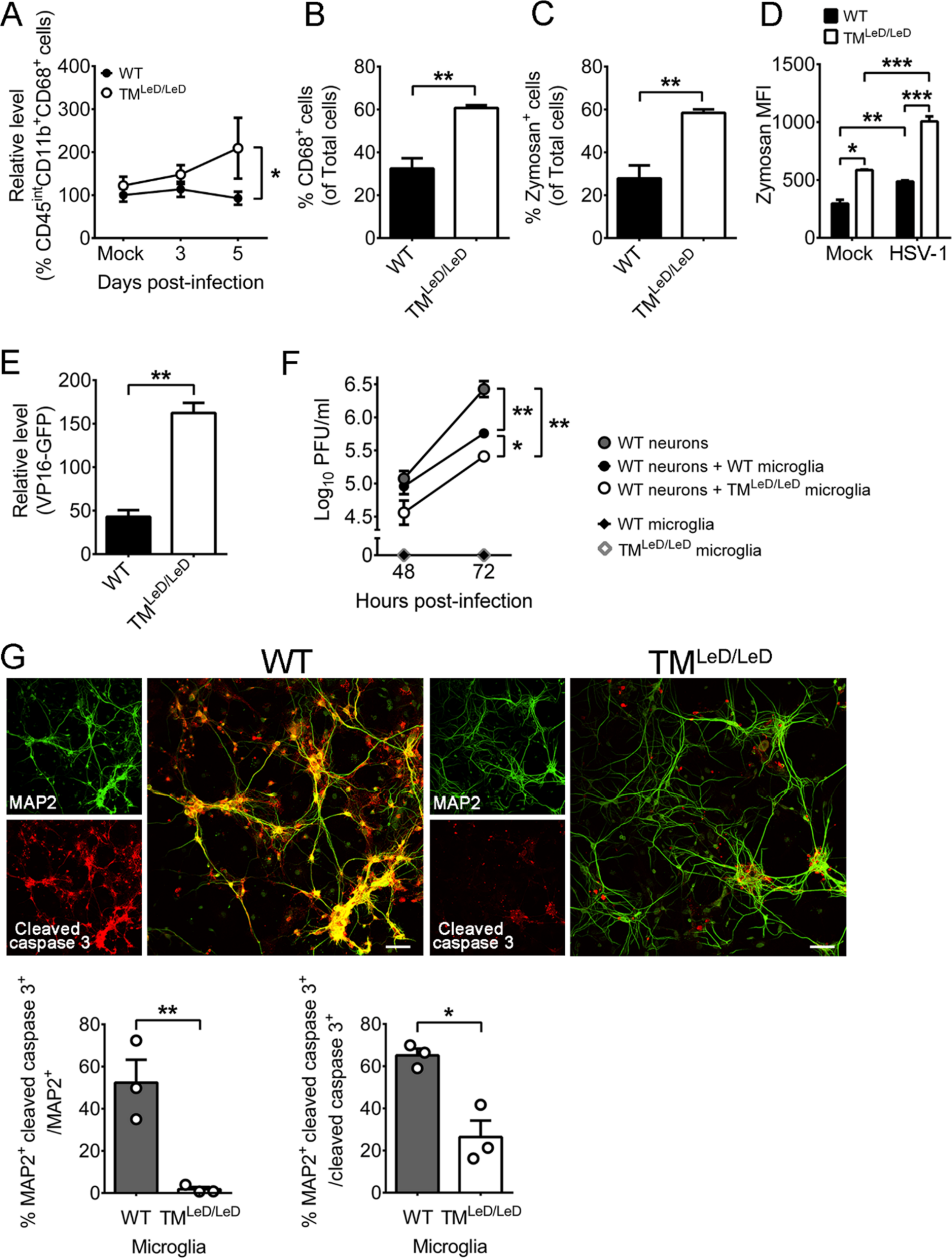
Absence of TM-LeD increases the microglia phagocytic activity and capacity to reduce HSV-1 replication in neurons. **A** Leukocytes purified from brains of mock-infected or infected WT or TM^LeD/LeD^ mice at 3 and 5 dpi were assayed for markers of microglia phagocytosis (CD45^int^CD11b^+^CD68^+^). The level of cells in mock-infected WT mice was set as 100%. Primary brain microglia cultured from WT or TM^LeD/LeD^ mice were assessed for CD68 (**B**) or subjected to the zymosan phagocytosis assay (**C**). **D** Primary brain microglia cultured from WT or TM^LeD/LeD^ mice were mock-infected or infected with HSV-1 (MOI = 0.001) for 23 h, incubated with zymosan for 1 h, and assayed for the mean fluorescence intensity (MFI). Panels **A**–**D** were assayed by flow cytometry. **E** Primary brain microglia cultured from WT or TM^LeD/LeD^ mice were incubated with HSV-1 strain DG-1 containing VP16-GFP (MOI = 10) for 30 min and assayed for GFP levels by a microplate reader. **F** Primary brain neurons were cultured from WT mice, incubated with or without primary brain microglia cultured from WT or TM^LeD/LeD^ mice, and infected with HSV-1 (MOI = 0.001). The whole infected culture with both cells and supernatant was harvested at the indicated hours for viral titration. **G** Samples prepared as described in **F** were subjected to immunofluorescence staining 72 hpi with antibodies against mouse MAP2 or cleaved caspase 3. Scale bar, 50 μm. MAP2^+^ and cleaved caspase 3^+^ cells in images (300 × 300 μm) were counted. The data represent means ± or + SEM (error bars) of 3–12 samples per data point or group. *P < 0.05; **P < 0.01; and ***P < 0.001

Microglia are professional phagocytes capable of eradicating virus by phagocytosis. CD68 is a phagosome/lysosome marker reflecting phagocytic activity [57]. We investigated the effect of TM-LeD on the phagocytic activity of microglia. In brains of mock-infected mice, the levels of (CD45^int^CD11b^+^CD68^+^) phagocytic microglia in WT and TM^LeD/LeD^ mice were similar (Fig. 4A). In brains of infected mice, the level of phagocytic microglia of WT mice was lower than that of TM^LeD/LeD^ mice at 5 dpi, but not at 3 dpi (Fig. 4A).

To further investigate the role of TM-LeD on microglial phagocytosis, we assayed primary microglia cultured from brains of neonatal WT or TM^LeD/LeD^ mice. The level of the phagocytic (CD68^+^) marker in microglia from WT mice was lower than that of microglia from TM^LeD/LeD^ mice as demonstrated by flow cytometry (Fig. 4B). To assess the phagocytic activity of microglia, we added green fluorescence-labeled zymosan, which can be phagocytosed and detected by flow cytometry. Under mock infection, the percentage of cells with fluorescence was lower in WT microglia than in TM^LeD/LeD^ microglia (Fig. 4C), and the mean fluorescence intensities (MFIs) of WT microglia was lower than that of TM^LeD/LeD^ microglia by twofold (Fig. 4D). We tested to infect microglia for 23 h before adding zymosan. The MFIs of infected WT and TM^LeD/LeD^ microglia were increased by 1.6 and 1.7-fold, respectively, when compared with their mock-infected controls (Fig. 4D). Under HSV-1 stimulation, the MFI level of WT microglia was lower than that of TM^LeD/LeD^ microglia by twofold (Fig. 4D). Collectively, our in vitro and in vivo studies reveal that TM-LeD suppresses the phagocytic activity of mouse brain microglia during HSV-1 infection (Fig. 4A–D). To determine the effect of TM-LeD on HSV-1 entry into brain microglia, we used recombinant (VP16-GFP) HSV-1 strain (DG-1), in which the virion contains GFP [25, 26]. Mouse primary brain microglia were infected with DG-1 for 30 min before treatment with citrate buffer to remove the virus outside cells. The fluorescence level of WT microglia was lower than that of TM^LeD/LeD^ microglia (Fig. 4E). Normally, particles, such as zymosan, that are bigger than 0.5 μm in diameter, can be phagocytosed [58, 59]. Although the size of HSV-1 virion is 0.2 μm [60, 61], HSV-1 virions associated with plasma membrane protrusions are shown to enter cells by phagocytosis-like uptake [62]. We monitored the replication of HSV-1 (strain DG or its parental, wild-type strain KOS) in microglia and found that both WT and TM^LeD/LeD^ microglia failed to support HSV-1 replication (with KOS results shown in Fig. 4F) in a manner consistent with previous reports of primary brain microglia cultured from mice or humans [63, 64]. These results showed that the high level of HSV-1 internalized into TM^LeD/LeD^ microglia was inactivated.

Human studies showed that neurons, the major HSV-1 target, are surrounded by microglia in the brain of patient with HSV-1 encephalitis [15, 57]. Our recent study using primary cells cultured from WT mouse brains showed that microglia reduced the levels of HSV-1 replication and apoptosis (cleaved caspase 3) of infected neurons [34]. Accordingly, we evaluated the role of microglial TM-LeD in reducing HSV-1 replication and damage of neurons. Primary neurons were cultured from brains of WT mouse embryos and infected with HSV-1, and the whole infected culture with both cells and supernatant was harvested to titrate virus. The virus titers increased from 48 to 72 h post-infection (hpi) (Fig. 4F), showing that mouse primary brain neurons support HSV-1 replication. We also co-cultured mouse primary brain microglia with neurons. Both WT and TM^LeD/LeD^ microglia decreased the levels of virus produced by neurons with TM^LeD/LeD^ microglia more efficiently than WT microglia (Fig. 4F). We monitored the apoptosis of neurons co-cultured with microglia and infected with virus using immunofluorescence staining with antibodies against cleaved caspase 3 or the neuron marker, MAP2. Co-culture of TM^LeD/LeD^ microglia significantly reduced the level of cleaved caspase 3 in neurons, when compared to WT microglia (Fig. 4G). Collectively, TM-LeD compromises the capacity of microglia to reduce HSV-1 replication and damage of neurons.

### In vitro studies using human cell lines show that TM-LeD knockdown increases microglia phagocytic and anti-HSV-1 activities

We examined whether TM-LeD-mediated suppression of microglia phagocytosis could be observed in human cells. To that end, we performed in vitro studies using the human brain microglia cell line, HMC3. As the antibody against TM domains 1–2, but not TM-LeD, is available commercially, we monitored TM and found that the mock-infected cells expressed TM and that HSV-1 infection increased TM expression in the cells within 4–12 hpi (Additional file 6: Fig. S4A, B). We also performed studies on the human neuronal cell line, SK-N-SH, in which TM was below detection in both mock-infected and infected cells (Additional file 6: Fig. S4D). TM was detected in 35% and 45% of brain microglia in mock-infected and infected mice, respectively (data not shown). In brain neurons, TM was below detection in both mock-infected and infected mice (data not shown). Accordingly, the results of human (HMC3 and SK-N-SH) cell lines and mouse brains are consistent.

**Fig. 5:**
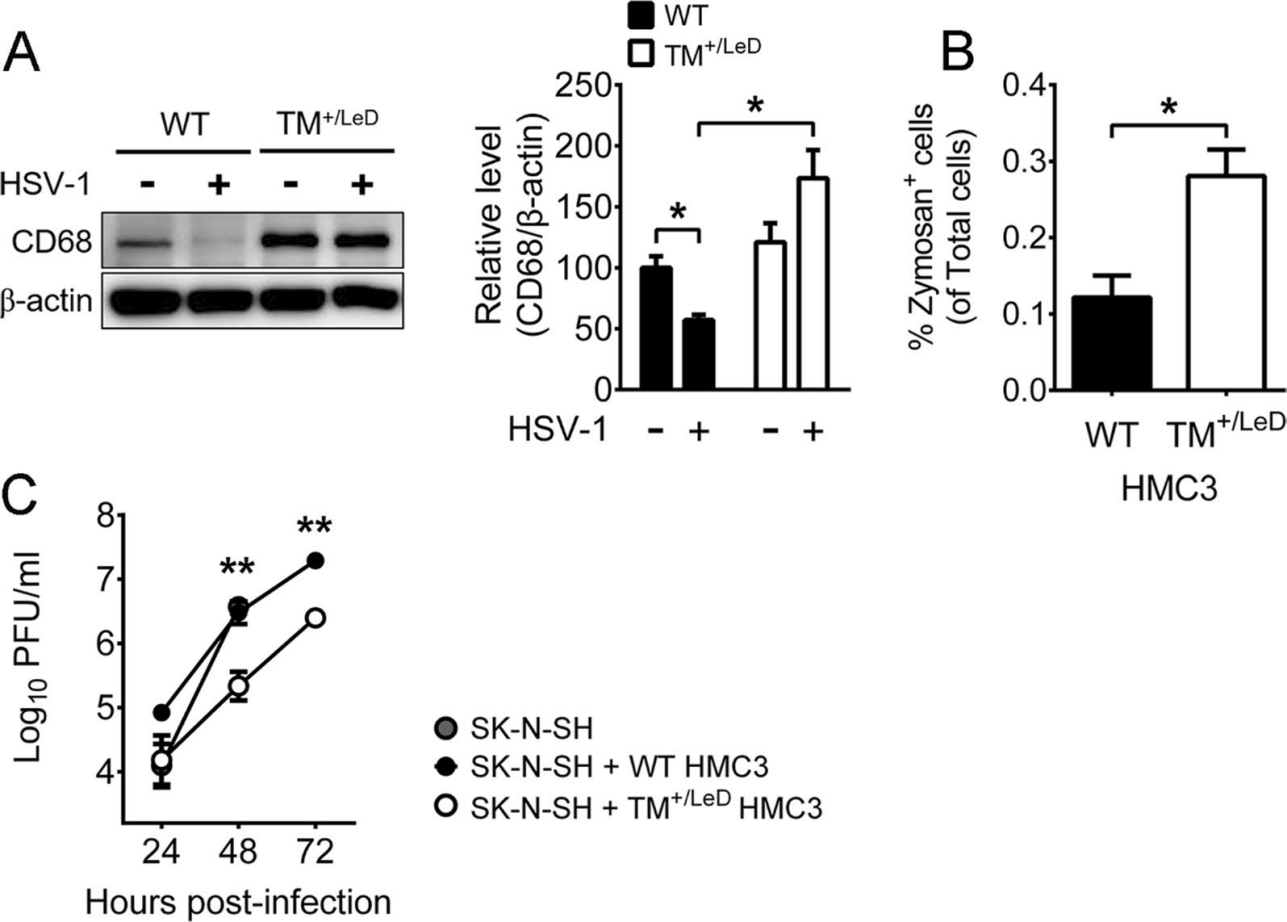
TM-LeD knockdown enhances the microglia phagocytic activity and capacity to reduce HSV-1 replication in neurons. **A** WT or TM^+/LeD^ HMC3 cells were mock-infected (–) or infected (+) with virus for 24 h (MOI = 1) and assayed for CD68 and β-actin by Western blotting. The levels of CD68 normalized to β-actin are shown. The mean of group with mock-infected (–) WT microglia only was set as 100%. **B** WT or TM^+/LeD^ HMC3 cells were infected with HSV-1 (MOI = 1) for 24 h and subjected to the zymosan phagocytosis assay. **C** SK-N-SH cells were co-cultured without or with WT or TM^+/LeD^ HMC3 cells, infected with HSV-1 (MOI = 0.001), and harvested at the indicated hours for viral titration. The data represent means ± or + SEM (error bars) of 3–7 samples per group or data point. *P < 0.05 and **P < 0.01

We used HMC3 cells to establish a human in vitro model of *TM-LeD* knockout by deleting the 465-base-pair coding sequence using the CRISPR/Cas9 system. After screening 80 cell clones, we found that 23 clones proliferated. Among these clones, there were six clones with heterozygous deletion (TM^+/LeD^), but there was no clone with homologous deletion. Among six TM^+/LeD^ clones, two clones, A and B, grew, but only clone B proliferated equally well as WT cells (data not shown). The TM level of WT cells was enhanced at 12 hpi (Additional file 6: Fig. S4A, B), but the TM levels of both clones A and B mock-infected or infected with HSV-1 for 12 h were below detection as demonstrated by Western blotting (Additional file 6: Fig. S4D). Clone B was used for further studies. To investigate the effect of TM-LeD knockdown on the phagocytic activity of human microglia in response to HSV-1 infection, we monitored CD68 using Western blotting. Under mock infection, the CD68 level of WT microglia was slightly lower than that of TM^+/LeD^ microglia (Fig. 5A). After HSV-1 infection, the CD68 level of WT cells was lower than that of TM^+/LeD^ cells (Fig. 5A). We further performed the zymosan phagocytosis assay and found that the level of zymosan^+^ cells in WT microglia was lower than that in TM^+/LeD^ microglia after HSV-1 infection (Fig. 5B). Accordingly, TM-LeD knockdown increases the phagocytic activity of human microglia.

To evaluate the effect of TM-LeD knockdown on the capacity for microglia to inhibit HSV-1 replication in human neuronal cells, SK-N-SH cells were co-cultured with or without HMC3 cells and infected with the virus. The virus titers of SK-N-SH cells were increased 24–72 hpi, showing that the cells support viral replication (Fig. 5C). Reduced virus titers were detected in SK-N-SH cells co-cultured with TM^+/LeD^ microglia, when compared to the co-culture with WT microglia, at 48 and 72 hpi (Fig. 5C). Collectively, our mouse in vivo and in vitro studies and human cell line study confirm that TM-LeD deficiency increases microglia phagocytic activities and the capacity of microglia to reduce HSV-1 replication in neurons.

## Discussion

Our in vivo study shows that absence of TM-LeD decreases the mortality, tissue viral loads, and brain neuron apoptosis of HSV-1-infected mice with increases in the number, proliferation, and phagocytic activity of brain microglia. In vitro studies reveal that mouse primary brain neurons cultured from WT or TM^LeD/LeD^ mice support viral replication to the same degree and that mouse primary brain microglia cultured from WT or TM^LeD/LeD^ mice suppress virus infectivity. Intriguingly, TM-LeD deficiency enhances the phagocytic activity of microglia. Moreover, co-culture of mouse primary brain microglia and neurons infected with HSV-1 shows that TM^LeD/LeD^ microglia decrease the level of virus produced by neurons more efficiently than WT microglia. These in vitro results showing that TM-LeD deficiency enhances the phagocytic activity of microglia to decrease the level of virus produced by neurons, explain the reduced levels of viral loads and antigens detected in brains of TM^LeD/LeD^ mice when compared to WT mice after infection. Here we identify TM-LeD to be the cellular factor aggravating HSV-1 encephalitis and provide the mechanism of action. We also demonstrate the significance of microglia and the phagocytic activity of microglia in HSV-1-induced encephalitis. In the brain of patients with HSV encephalitis, infected neurons are surrounded by microglia with phagocytic activity [57]. Our study suggests that microglia may suppress viral replication and damage of neurons and exert antiviral activity by phagocytosis in the brain.

Our recent mouse report showed that HSV-1 infection increases the number of microglia to outnumber those of infiltrating leukocytes (macrophages, neutrophils, and T cells) in the brain [34]. Moreover, HSV-1 infection increases the activation (phagocytic activity) of microglia as demonstrated by the expression of CD68. Depletion of microglia increases HSV-1 lethality, tissue viral loads, and brain neuron loss of infected mice. Regarding the protective mechanism, in vitro studies showed that microglia can reduce HSV-1 directly and indirectly. Microglia harvested from infected mice reduce virus infectivity. Microglia induce IFN-β, which in turn activates STAT1 to inhibit viral replication and damage in neurons during co-culture of both cells. The present study is novel in showing the regulation of TM-LeD on brain microglia and virus infection, as TM-LeD deficiency reduces HSV-1 lethality, tissue viral loads, and brain neuron damage of infected mice with increases in the number, proliferation, and phagocytic activity of microglia.

Thrombin is shown to increase endothelial and smooth muscle cell proliferation, and TM reduces the proliferation of endothelial cells and smooth muscle cells induced by thrombin [65, 66]. In addition, TM reduces the proliferation of tumor cells in a mechanism independent of thrombin [67]. Few studies investigate the effect of TM on leukocyte proliferation. Absence of TM-LeD is shown to reduce neutrophil influx into the lung of mice infected with *Streptococcus pneumonia* and to decrease monocyte/macrophage infiltration into the synovium of mice with arthritis [11, 14]. However, absence of TM-LeD is reported to increase neutrophil influx into the lung of LPS-treated mice as well as the heart of mice with ischemia and to enhance the infiltration of neutrophils and monocytes/macrophages into the kidney of mice treated with LPS and Shiga toxin [9, 12, 13]. These studies show the conflicting role of TM-LeD in regulating the numbers of neutrophils, monocytes, and macrophages in mouse tissues in various disease models. Absence of TM-LeD is found to enhance the expression of leukocyte adhesion molecules to promote neutrophil influx into the lung of LPS-treated mice and the infiltration of neutrophils and monocytes/macrophages into the kidney of mice treated with LPS and Shiga toxin [9, 12]. Few studies investigate whether TM-LeD modulates the proliferation of neutrophils, monocytes, and macrophages. Very few studies report the influence of TM-LeD on microglia. Here we showed that absence of TM-LeD increases microglia proliferation in mock-infected and infected mice and the phagocytic activity (CD68 expression) of microglia in infected mice. The signal molecule, Akt is upstream of mTOR, which is followed by NF-κB [68], and the axis of Akt–mTOR–NF-κB is demonstrated to regulate cell proliferation, microglia phagocytosis, and/or microglia CD68 expression [68–70]. Both endogenous TM-LeD and exogenous TM-LeD treatment inhibit the signaling of NF-κB and MAPK to diminish the immune response, such as cytokine production, of non-microglia after LPS challenge [2, 9, 45, 46]. Exogenous TM treatment suppresses autophagy activation in endothelial cells by promoting the mTOR/Akt pathway [71]. Microglia exert anti-HSV-1 activity by producing antiviral mediators, IFNs to activate the signaling of STAT1 and STAT3 [48–50, 72, 73]. We found that TM-LeD fails to affect the responses of cytokines regulated by NF-κB and MAPK and IFN responses in mice and brain microglia from infected mice. Moreover, our unpublished results showed that absence of TM-LeD failed to affect the activation of mTOR, Akt, NF-κB, STAT1, and STAT3 in mouse primary brain microglia co-cultured with or without neurons and infected with or without HSV-1. This suggests that in our model, TM-LeD hampers the immune responses of brain microglia by the novel signaling pathway independent of MAPK, mTOR, Akt, NF-κB, STAT1, and STAT3. Future studies are needed to identify the signaling pathway mediated by TM-LeD to suppress microglia proliferation in mock-infected and HSV-1-infected mice and to reduce the phagocytic activity of microglia in infected mice.

In addition to the traditional microglia markers (CD45^int^CD11b^+^), Tmem119, a microglia-specific marker [42], was also used to characterize and quantify microglia in our study. However, a recent study showed that the expression of Tmem119 was reduced in microglia during activation [74]. This explains the results obtained in mouse brains. In brains of mock-infected mice, as TM-LeD deficiency slightly expands the number of activated microglia with phagocytic activity (CD45^int^CD11b^+^CD68^+^ cells), TM-LeD deficiency is found to increase the number CD45^int^CD11b^+^ microglia, but not CD45^+^CD11b^+^Tmem119^+^ microglia. In brains of infected mice, TM-LeD deficiency increases the numbers of CD45^int^CD11b^+^ microglia at both 3 and 5 dpi and the number of CD45^+^CD11b^+^Tmem119^+^ microglia only at 3 dpi, as TM-LeD deficiency amplifies the number of activated microglia with phagocytic activity (CD45^int^CD11b^+^CD68^+^ cells) at 5 dpi. We used Ki67, which is present mostly in growing and dividing cells under G1, S, G2, and M phases, to assess brain microglia [75]. Cells in G0 phase, like most of resting microglia, the Ki67 level is low. In brains of infected mice, more CD45^+^CD11b^+^Tmem119^+^Ki67^+^ microglia were detected in TM^LeD/LeD^ mice than in WT mice at 3 dpi. This result indicates that TM-LeD deficiency promotes the proliferation potential of microglia in infected mice and could explain the abundant CD45^+^CD11b^+^Tmem119^+^ microglia detected in infected TM^LeD/LeD^ mice, when compared to infected mice, at 3 dpi. However, Ki67^+^ cells could remain in G1 phase and fail to complete cell cycle and duplicate due to the lack of further stimulation, such as HSV-1 infection. This may result in the discrepancy found in brains of mock-infected mice with more CD45^+^CD11b^+^Tmem119^+^Ki67^+^ microglia, but not CD45^+^CD11b^+^Tmem119^+^ microglia, detected in TM^LeD/LeD^ mice than in WT mice. The results of using different markers (CD45^int^CD11b^+^, Tmem119^+^, and Ki67^+^) to characterize microglia show that the expression of Tmem119 is decreased during microglia activation/proliferation and that TM-LeD deficiency increases the number and proliferation of microglia, especially in infected mice.

In humans, the gender affects the serum TM level, which is significantly lower in the female group than in the male group of patients with atrial fibrillation, the disease with female gender to be one of the risk factors [76]. We used female mice for studies, because we found comparable mortality rates (50%) in infected male WT and TM^LeD/LeD^ mice (*n* = 4 and 6, respectively). Future studies using a large sample size of mice and testing different viral doses should be performed on male mice to determine whether and how the gender affects the regulation of TM-LeD on HSV-1 lethality and whether the gender affects the TM-LeD level. In humans, there are few reports on the association of gender with HSV encephalitis, and the correlation of the TM-LeD level and HSV encephalitis can be studied in future.

CD4 T cells are shown to protect mice from HSV-1 infection, as mice lacking CD4 T cells are susceptible to virus-induced encephalitis, when compared to WT mice [77]. However, some subsets of CD4 T cells have been shown to promote the HSV disease and infection in mice. Regulatory T cells are found to reduce the antiviral activity of CD8 T cells to promote HSV-1 reactivation from latency in mouse trigeminal ganglia [78], and T_H_2 cells are shown to aggravate the severity of encephalomyelitis in HSV-2-infected mice [79]. We showed that the number of CD4 T cells in brains of infected WT mice is higher than that of infected TM^LeD/LeD^ mice. Nevertheless, the increase of CD4 T cells fails to compensate for the loss of microglia to reduce the death and brain viral loads of infected WT mice. These results highlight the significance of microglia in anti-HSV-1 immunity when compared to CD4 T cells. We further analyzed CD4 T cells and found that the numbers of regulatory T cells and T_H_2 cells in brains of infected WT mice were significantly and slightly higher than those of infected TM^LeD/LeD^ mice, respectively. The increase of regulatory T cells may compromise the anti-HSV-1 immunity in brains of infected WT mice.

Endogenous TM-LeD is found to protect mice against the inflammation induced by LPS alone or plus Shiga toxin, myocardial ischemia, and arthritis [9, 12–14]. Treatment with human TM-LeD can protect mice against arthritis, LPS, Gram^−^ bacteria (*Klebsiella pneumonia*), UV irradiation–induced skin injury, and atherosclerosis [14, 45, 46, 80]. Thus, human TM domains 1–3, which protects mice from LPS [81, 82], was tested in the phase 3 clinical trial for patients with severe sepsis [81, 82]. We find that absence of TM-LeD decreases HSV-1 lethality in mice with increases in the number and phagocytic activity of brain microglia and anticipate that suppression of TM-LeD may boost the brain microglia number and phagocytic activity to reduce HSV-1 lethality. For a molecule to cross the blood–brain barrier, the molecular mass must be under 400–500-Da [83]. TM-LeD, with 155 amino acids and the molecular weight of 35 kDa [45], currently used to reduce various models of diseases in periphery is too large to penetrate into the brain efficiently. Future studies should be designated to design and test small molecules against TM-LeD to reduce HSV-1-indeuced encephalitis in the infected host.

## Conclusions

Collectively, we demonstrate the novel cellular factor, TM-LeD, which down regulates the proliferation and phagocytic activity of brain microglia in HSV-1-infected mice. Our report provides the pathogenic mechanism of TM-LeD in HSV-1-indiced encephalitis and the potential target for the development of therapeutics to reduce the disease.

## Supplementary Information

**Additional file 1:** RNA isolation and quantitative RT-PCR.

**Additional file 2: Table S1.** Primer sequences for RT-PCR.

**Additional file 3: Figure S1.** Absence of TM-LeD fails to affect HSV-1 replication in mouse primary cells.

**Additional file 4: Figure S2.** Effects of TM-LeD on leukocytes in the brains of infected mice.

**Additional file 5: Figure S3.** Effects of TM-LeD on the cytokine levels in the brains or microglia of infected mice.

**Additional file 6: Figure S4.** HSV-1 infection enhances TM expression in the human microglia cell line, HMC3.

## Data Availability

Share upon reasonable request.

## Abbreviations

Akt: Protein kinase B
CD: Cluster of differentiation
Cxcl10: C-X-C motif chemokine 10
Gram^−^: Gram-negative
HMGB1: High mobility group box 1
HSV-1: Herpes simplex virus 1
IFN: Interferon
IL: Interleukin
iNOS: Inducible NO synthase
ISG: Interferon-stimulated gene
LeD: Lectin-like domain
LPS: Lipopolysaccharides
MAPK: Mitogen-activated protein kinase
MEFs: Mouse embryonic fibroblasts
MFI: Mean fluorescence intensity
MOI: Multiplicity of infection
mTOR: Mammalian target of rapamycin
Mx1: MX dynamin-like GTPase 1
PFU: Plaque forming unites
dpi: Days post-infection
hpi: Hours post-infection
RAGE: Receptor for advanced glycation end-products
STAT: Signal transducer and activator of transcription 1
TM: Thrombomodulin
TNF: Tumor necrosis factor
WT: Wild type
