# Supplementary material for: Absence of the lectin-like domain of thrombomodulin reduces HSV-1 lethality of mice with increased microglia responses
Source: J Neuroinflammation. 2022 Mar 11;19:66. doi: 10.1186/s12974-022-02426-w (PMC8915510; doi:10.1186/s12974-022-02426-w)
Supplement: Supplementary file 1 — Additional file 1: RNA isolation and quantitative RT-PCR. [file 12974_2022_2426_MOESM1_ESM.docx]

**Additional material and methods**

**RNA isolation and quantitative RT-PCR**

Cells were frozen, and total RNA was extracted by the RNeasy lipid tissue mini kit (Qiagen). After reverse transcription with reverse primers, the synthesized cDNA was used for quantitative PCR with the forward and reverse primers listed in Additional Table S1. PCR was performed with initial activation for 10 min at 95°C followed by 40 cycles of denaturation (95°C, 15 seconds) and annealing (60°C, 1 min) with the kit of Fast SYBR Green Master Mix (Thermo Fisher Scientific). The threshold cycle (*C*_T_) of each product was determined, normalized to the internal control β-actin, and shown as Δ*C*_T_. All results are shown as a ratio to β-actin calculated as 2^-Δ^*^C^*^T^.
