# Supplementary material for: Absence of the lectin-like domain of thrombomodulin reduces HSV-1 lethality of mice with increased microglia responses
Source: J Neuroinflammation. 2022 Mar 11;19:66. doi: 10.1186/s12974-022-02426-w (PMC8915510; doi:10.1186/s12974-022-02426-w)
Supplement: Supplementary file 2 — Additional file 2: Table S1. Primer sequences for RT-PCR. [file 12974_2022_2426_MOESM2_ESM.docx]

**Additional Table S1. Primer sequences for RT-PCR**

| Genes | Forward Primer (5’-3’) | Reverse primer (5’-3’) |
| --- | --- | --- |
| *Ifnb* | GGTGGAATGAGACTATTGTTG | AGGACATCTCCCACGTC |
| *Ifng* | TTCTTCAGCAACAGCAAGGC | TCAGCAGCGACTCCTTTTCC |
| *Ifnl* | AGCTGCAGGTCCAAGAGCG | GGTGGTCAGGGCTGAGTCATT |
| *Cxcl10* | GCTGGGATTCACCTCAAGAA | CTTGGGGACACCTTTTAGCA |
| *Mx1* | CTGAGATGACCCAGCACCTGAA | CTCCAGGAACCAGCTGCACTTAC |
| *Nos2* | CAGCTGGGCTGTACAAACCTT | CATTGGAAGTGAAGCGTTTCG |
| *Il1b* | GCAAGTGTCTGAAGCAGCTATG | CCACAGCCACAATGAGTGATAC |
| *Il6* | CCTCTGGTCTTCTGGAGTACC | ACTCCTTCTGTGACTCCAGC |
| *Tnf* | ATGAGCACAGAAAGCATGA | AGTAGACAGAAGAGCGTGGT |
| *Arg1* | CTCCAAGCCAAAGTCCTTAGAG | AGGAGCTGTCATTAGGGACATC |
| *Il10* | ATAACTGCACCCACTTCCCA | GGGCATCACTTCTACCAGGT |
| *Tgfb* | CCTGCAAGACCATCGACATG | TGTTGTACAAAGCGAGCACC |
| *Actb* | AACCCTAAGGCCAACCGTGAAAAGATGACC | CCAGGGAGGAAGAGGATGCGGC |
