# Supplementary material for: Absence of the lectin-like domain of thrombomodulin reduces HSV-1 lethality of mice with increased microglia responses
Source: J Neuroinflammation. 2022 Mar 11;19:66. doi: 10.1186/s12974-022-02426-w (PMC8915510; doi:10.1186/s12974-022-02426-w)
Supplement: Supplementary file 3 — Additional file 3: Figure S1. Absence of TM-LeD fails to affect HSV-1 replication in mouse primary cells. [file 12974_2022_2426_MOESM3_ESM.docx]

**
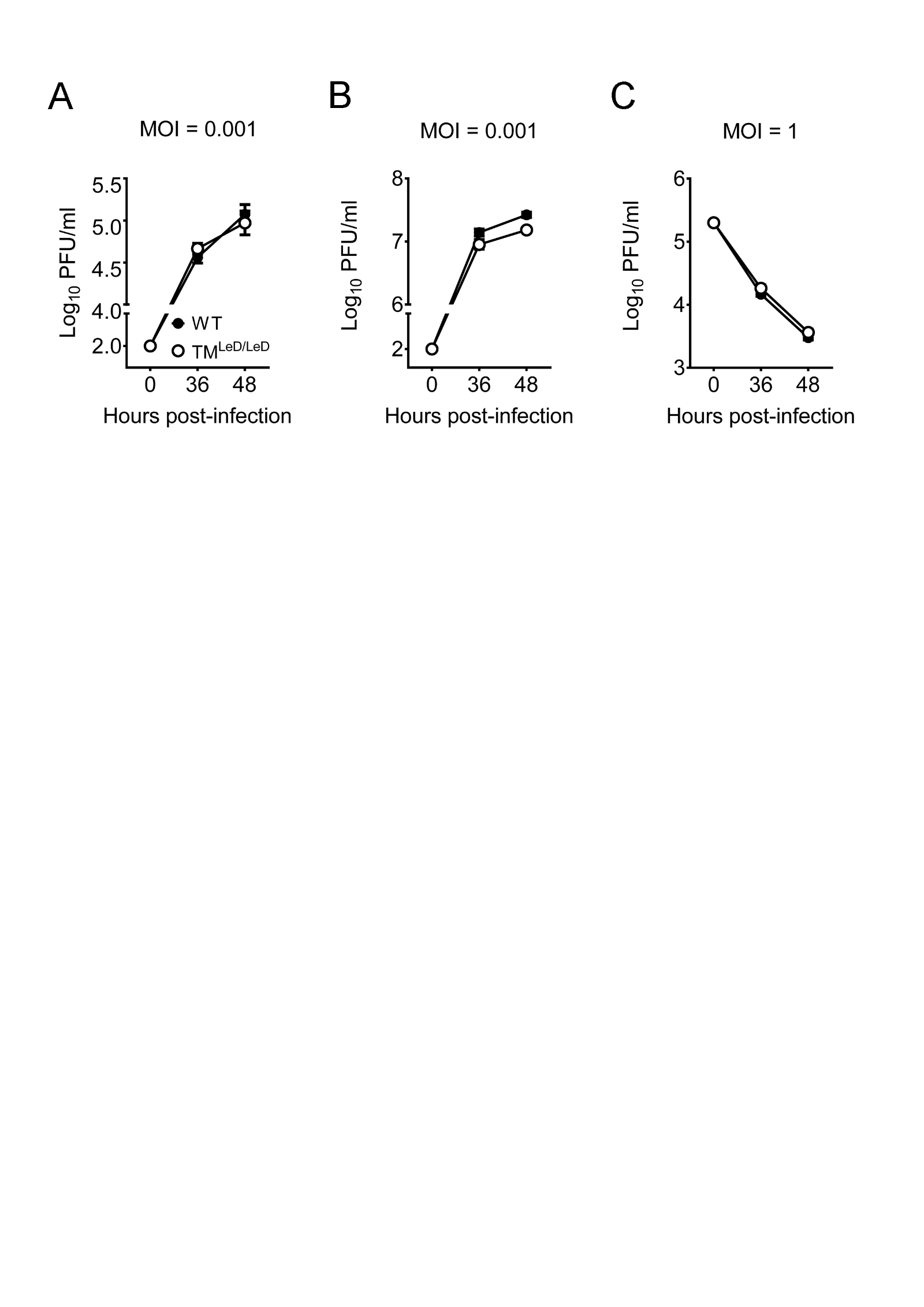
**

**Additional Figure S1.** **Absence of TM-LeD fails to affect HSV-1 replication in mouse primary cells.** (A) Primary brain neurons, (B) embryonic fibroblasts, and (C) brain microglia cultured from WT or TM^LeD/LeD^ mice were infected with HSV-1 at the indicated MOIs and harvested at the indicated hours to determine viral titers. The data represent means ± SEM (error bars) of >3 sample per data point.
