# Supplementary material for: Absence of the lectin-like domain of thrombomodulin reduces HSV-1 lethality of mice with increased microglia responses
Source: J Neuroinflammation. 2022 Mar 11;19:66. doi: 10.1186/s12974-022-02426-w (PMC8915510; doi:10.1186/s12974-022-02426-w)
Supplement: Supplementary file 4 — Additional file 4: Figure S2. Effects of TM-LeD on leukocytes in the brains of infected mice. [file 12974_2022_2426_MOESM4_ESM.docx]

**
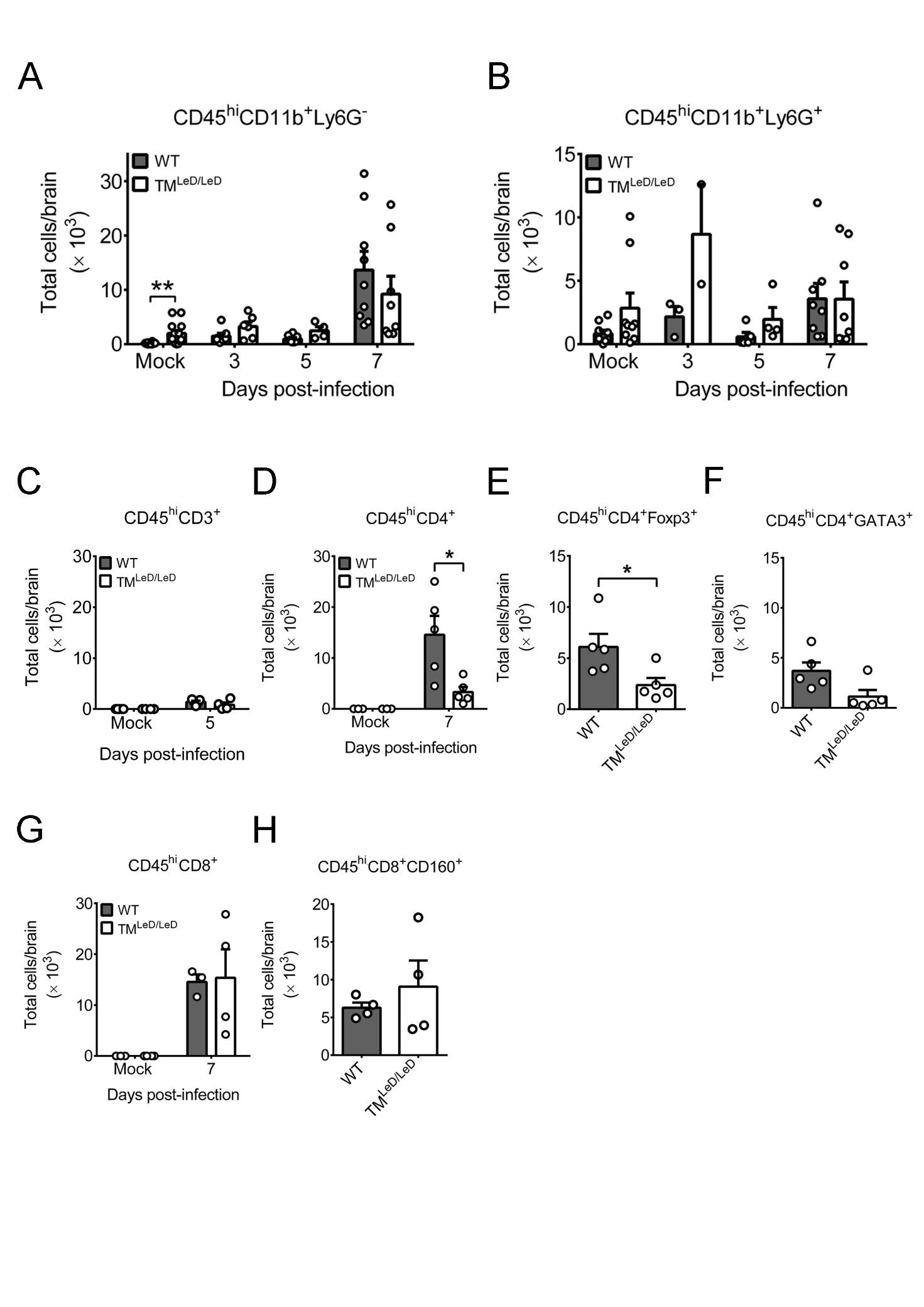
**

**Additional Figure S2.** **Effects of TM-LeD on leukocytes in the brains of infected mice.** Leukocytes purified from the brains of WT and TM^LeD/LeD^ mice mock-infected or infected with HSV-1 were assayed for markers of (A) (CD45^hi^CD11b^+^Ly6G^-^) macrophages, (B) (CD45^hi^CD11b^+^Ly6G^+^) neutrophils, (C) (CD45^hi^CD3^+^) T cells, (D) (CD45^hi^CD4^+^) CD4 T cells, (E) (CD45^hi^CD4^+^Foxp3^+^) regulatory T cells, (F) (CD45^hi^CD4^+^GATA3^+^) T_H_2 cells, (G) (CD45^hi^CD8^+^) CD8 T cells, and (H) (CD45^hi^CD8^+^CD160^+^) activated CD8 T cells by flow cytometry. Antibodies against mouse, CD4 (Clone GK-1.5; BioLegend), CD8 (Clone 53-6.7; BioLegend), CD160 (Clone 7H1; BioLegend), Foxp3 (Clone MF23; BD Biosciences), or GATA3 (Clone 16E10A23; BioLegend) were used. Brains were harvested from infected mice on the indicated days (A-D, G) or on day 7 post-infection (E, F, H). The data represent means + SEM (error bars) of ≥2 samples per group. *, *P* < 0.05 and **, *P* < 0.01, via a Mann-Whitney *U* test.
