# Supplementary material for: Absence of the lectin-like domain of thrombomodulin reduces HSV-1 lethality of mice with increased microglia responses
Source: J Neuroinflammation. 2022 Mar 11;19:66. doi: 10.1186/s12974-022-02426-w (PMC8915510; doi:10.1186/s12974-022-02426-w)
Supplement: Supplementary file 5 — Additional file 5: Figure S3. Effects of TM-LeD on the cytokine levels in the brains or microglia of infected mice. [file 12974_2022_2426_MOESM5_ESM.docx]

**
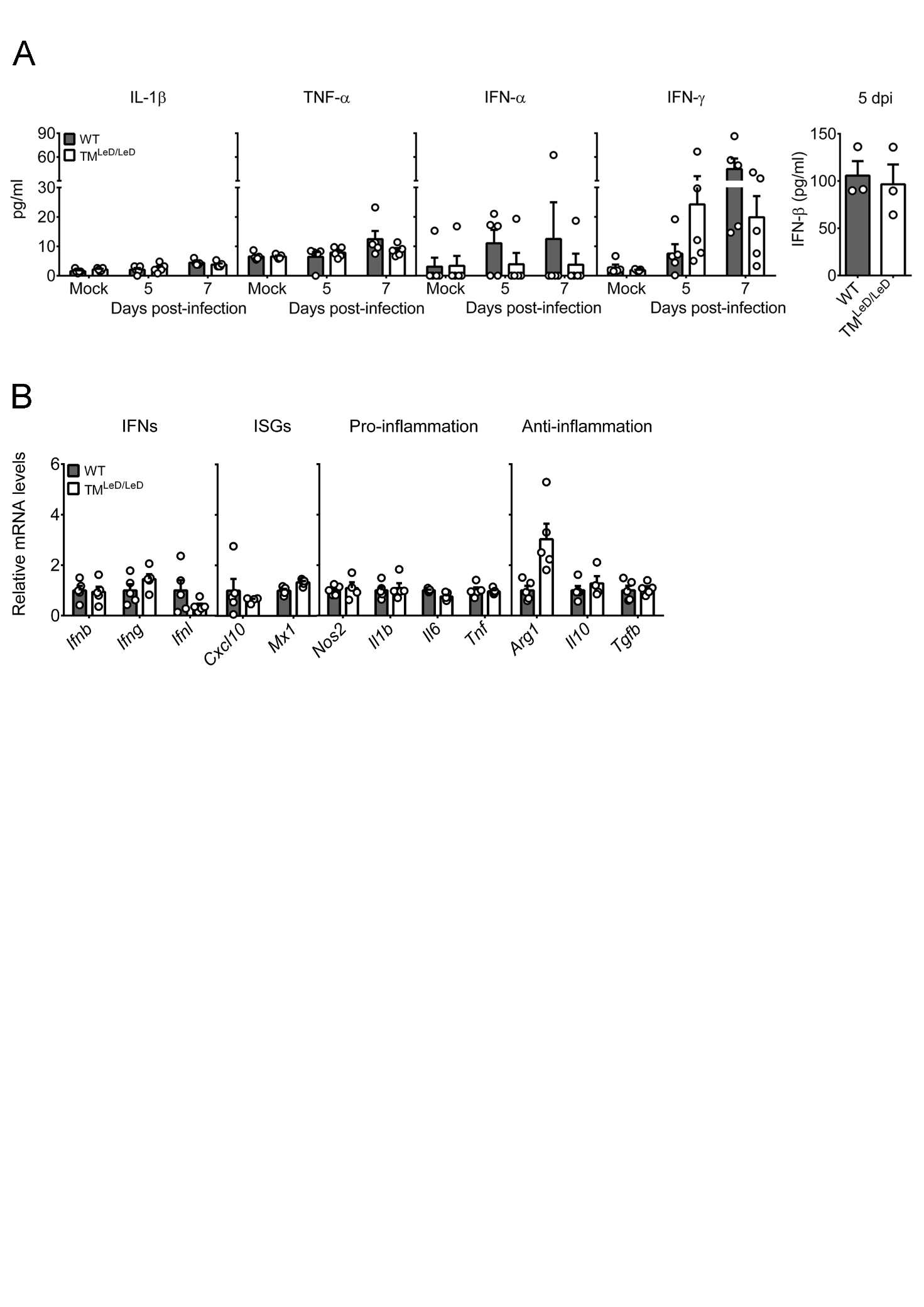
**

**Additional Figure S3.** **Effects of TM-LeD on the cytokine levels in the brains or microglia of infected mice.** (A) The brains of WT and TM^LeD/LeD^ mice mock-infected or infected with HSV-1 were harvested at 5 and/or 7 dpi and assayed for the indicated cytokines by Luminex or for IFN-β by ELISA (BioLegend). (B) Brains of infected WT or TM^LeD/LeD^ mice were harvested at 5dpi. Microglia were isolated from brains by the MojoSort^TM^ mouse P2RY12 selection kit (BioLegend) and assayed for the indicated immune modulators by quantitative RT-PCR. The mRNA level of indicated gene was normalized to that of *β-actin* in each sample. The mean mRNA levels of indicated immune modulators of infected WT mice are set as 1. The data represent means + SEM (error bars) of 3-5 samples per group.
