# Supplementary material for: Absence of the lectin-like domain of thrombomodulin reduces HSV-1 lethality of mice with increased microglia responses
Source: J Neuroinflammation. 2022 Mar 11;19:66. doi: 10.1186/s12974-022-02426-w (PMC8915510; doi:10.1186/s12974-022-02426-w)
Supplement: Supplementary file 6 — Additional file 6: Figure S4. HSV-1 infection enhances TM expression in the human microglia cell line, HMC3. [file 12974_2022_2426_MOESM6_ESM.docx]

**
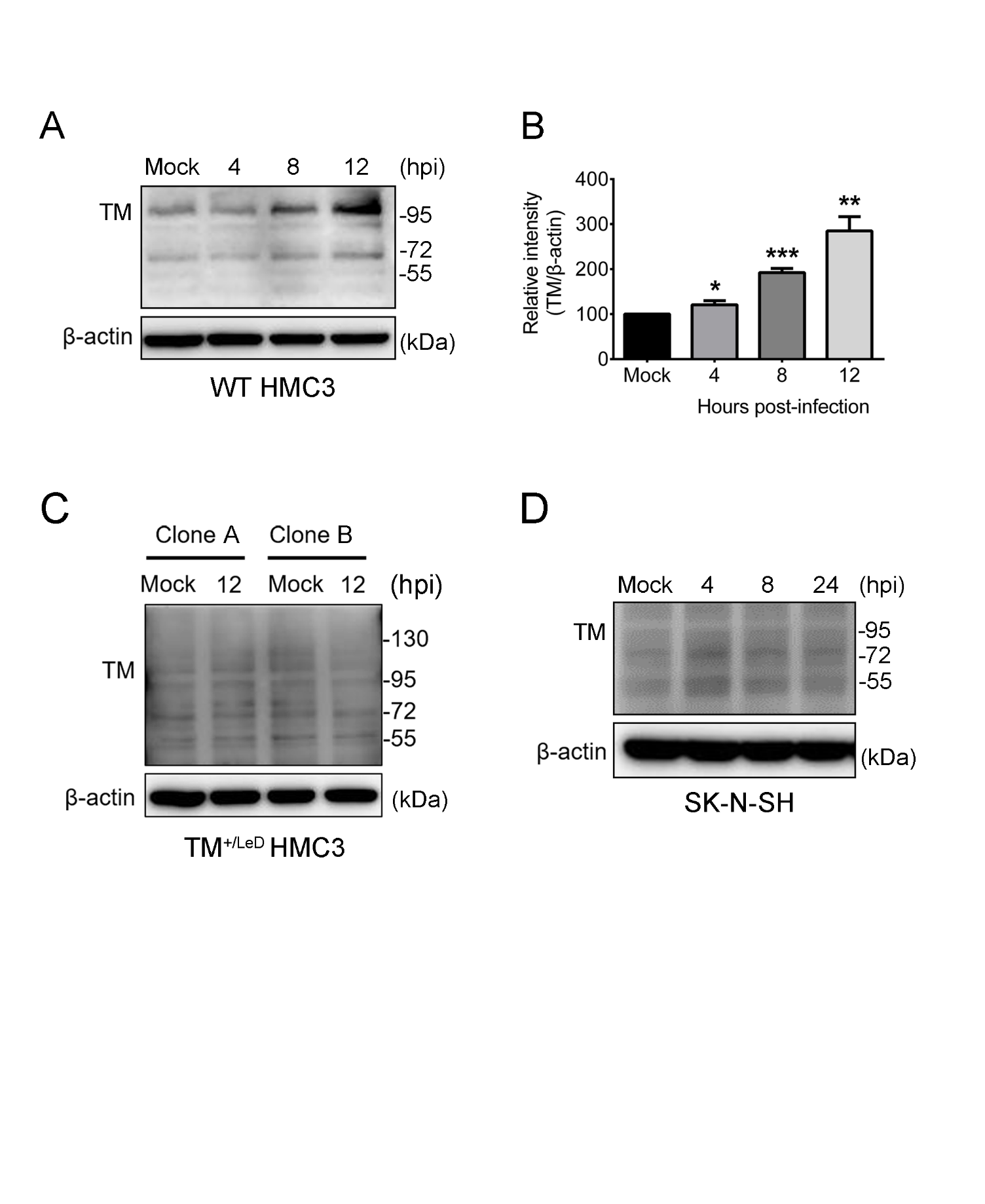
**

**Additional Figure S4.** **HSV-1 infection enhances TM expression in the human microglia cell line, HMC3.** (A-B) WT HMC3, (C) HMC3 with TM-LeD knockdown (TM^+/LeD^), and (D) SK-N-SH were mock-infected or infected with HSV-1 (MOI = 1), harvested at the indicated hpi, and assayed for TM with the antibody against amino acids 22-321 (domains 1 to 2) of TM (Clone D-3; Santa Cruz Biotechnology) by Western blotting. (B) For each sample, the level of TM band was normalized to that of β-actin band. The mean of mock-infected group is set as 100%. The data represent means + SEM (error bars) of ≥3 samples per group. *, *P* < 0.05; **, *P* < 0.01; and ***, *P* < 0.001, via a Student *t* test.
